# Supplementary material for: Thermoplastic Elastomer (TPE)–Poly(Methyl Methacrylate) (PMMA) Hybrid Devices for Active Pumping PDMS-Free Organ-on-a-Chip Systems
Source: Biosensors (Basel). 2021 May 19;11(5):162. doi: 10.3390/bios11050162 (PMC8160665; doi:10.3390/bios11050162)
Supplement: Supplementary file 1 [file biosensors-11-00162-s001.zip › biosensors-1217991-sup/Suppl.pdf]

Supplementary Material

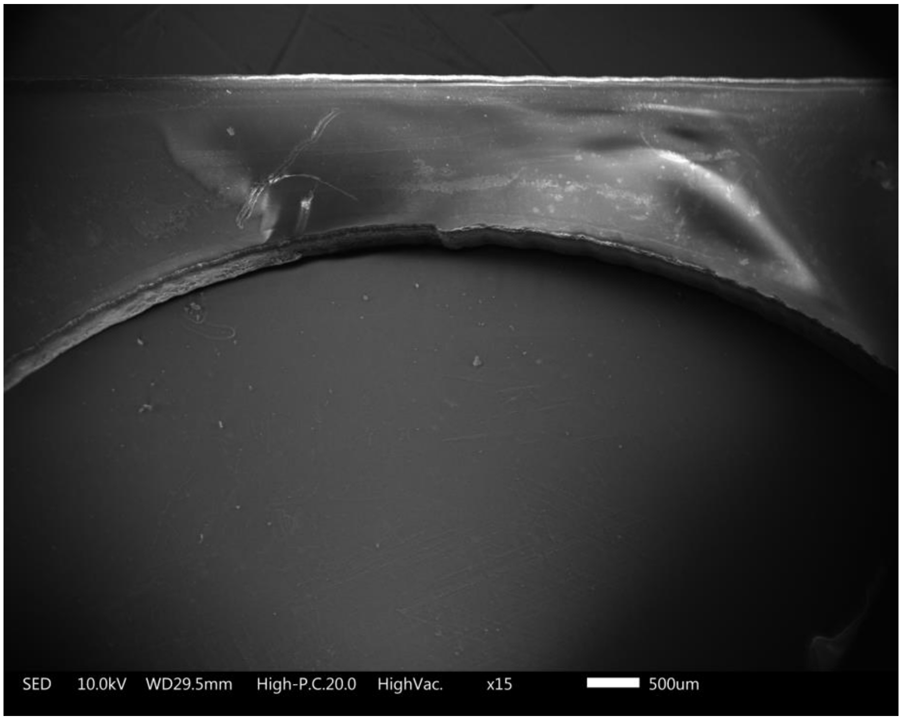

Fig. S1: SEM image of a laser-cut 250 um PMMA film bonded to a 1.5 mm PMMA sheet, imaged after pressure testing (delamination)

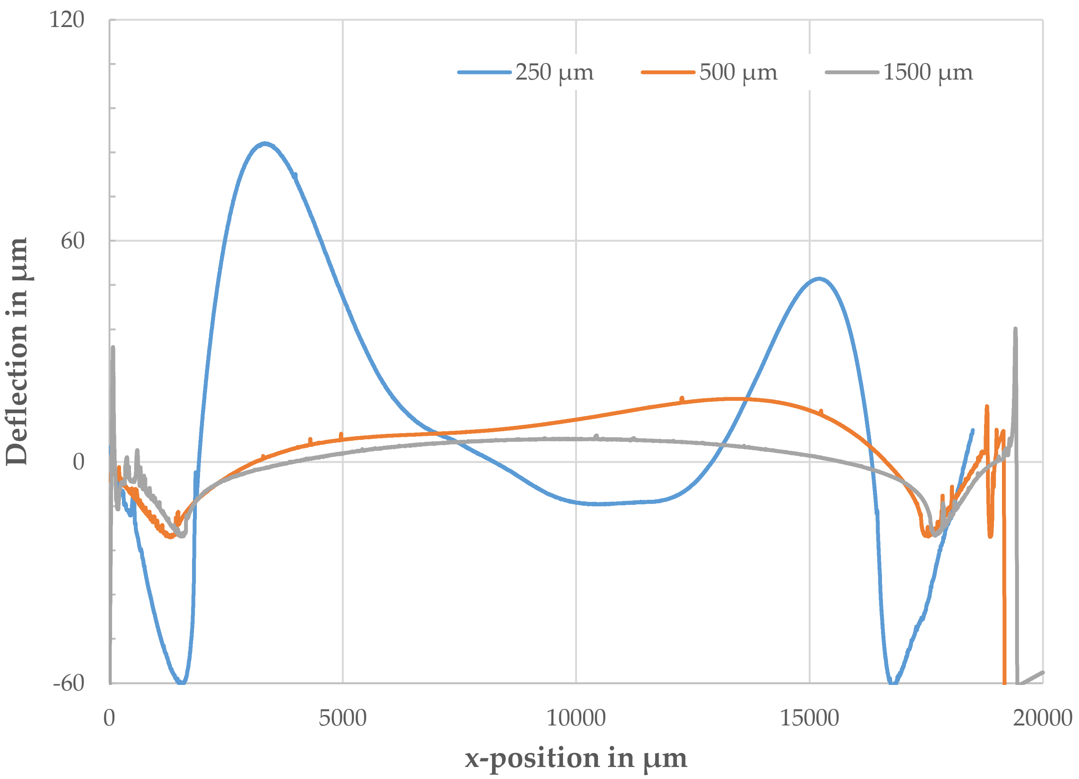

Fig. S2: Distortion of different thick PMMA substrate after thermal bonding (pressure: 2.4 MPa, temperature: 84 °C, UV dose: 3 J/cm2). Samples were obtained after pressure testing and deflection was measured with a profilometer (line-scan).

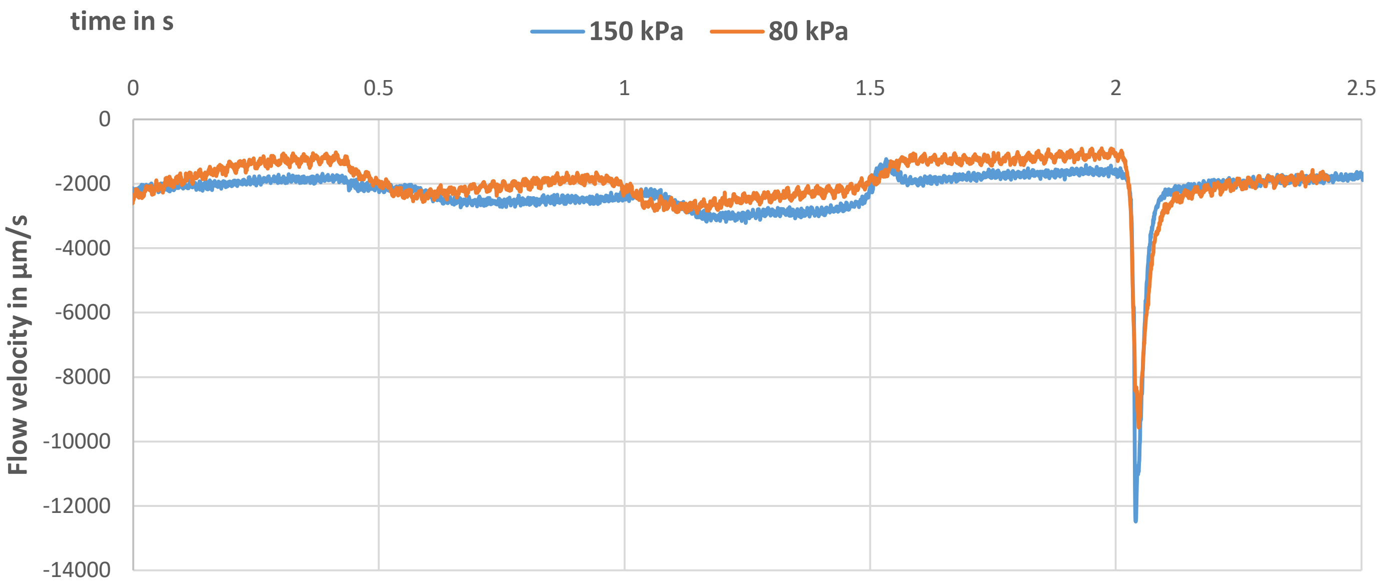

Fig. S 3: Velocity time curves for a driving pressure of 80 and 150 kPa at an actuation frequency of 1.2 Hz and a driving vacuum of -80 kPa. Measured in a 1 mm wide and 0.5 mm high channel.

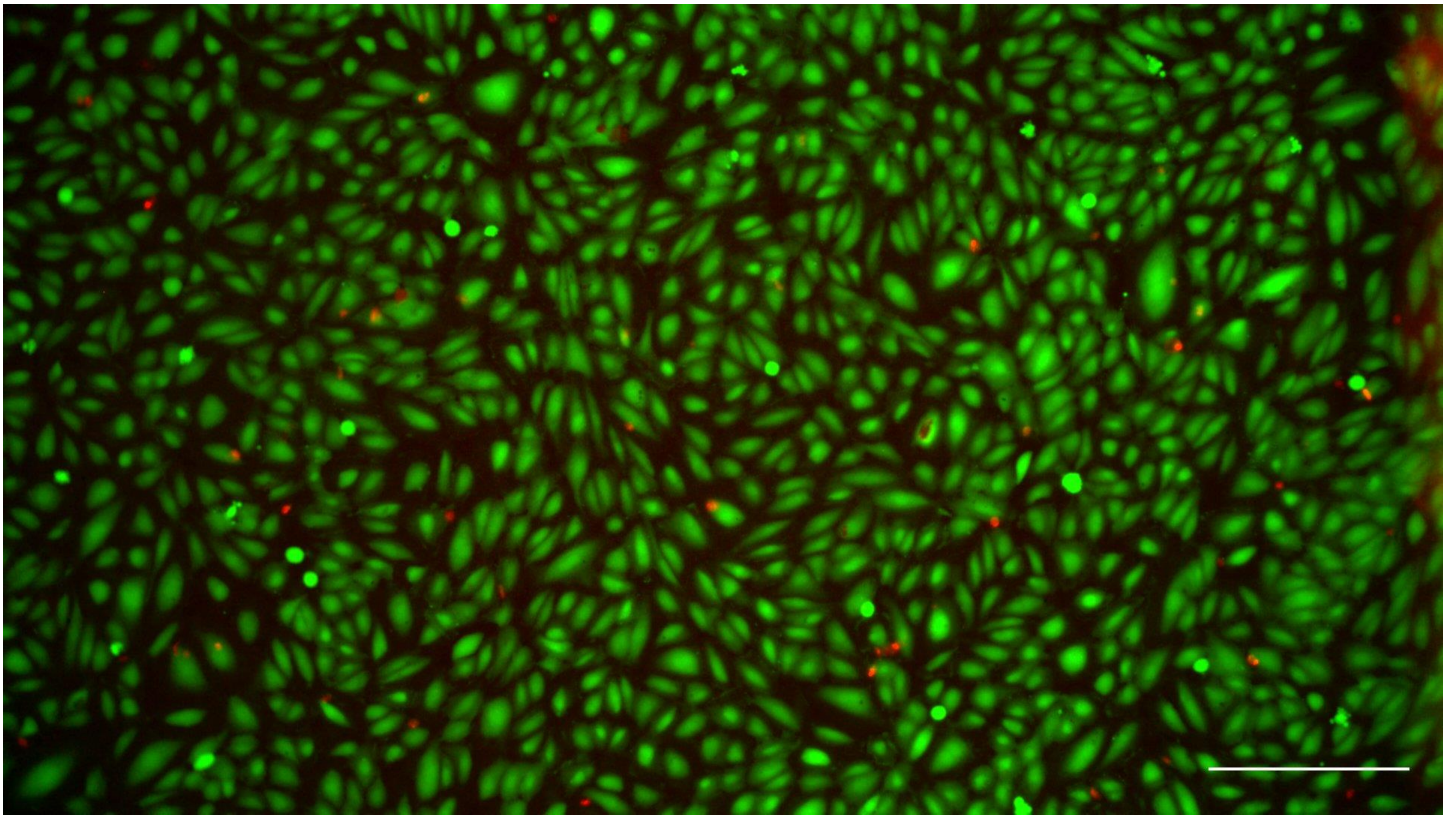

Fig. S 4: Live-dead staining of HUVEC cells after 24h cultivation on-chip (control: perfusion on tilting platform). Green: living cells. Red: Dead cells. Scale bar: 200 µm

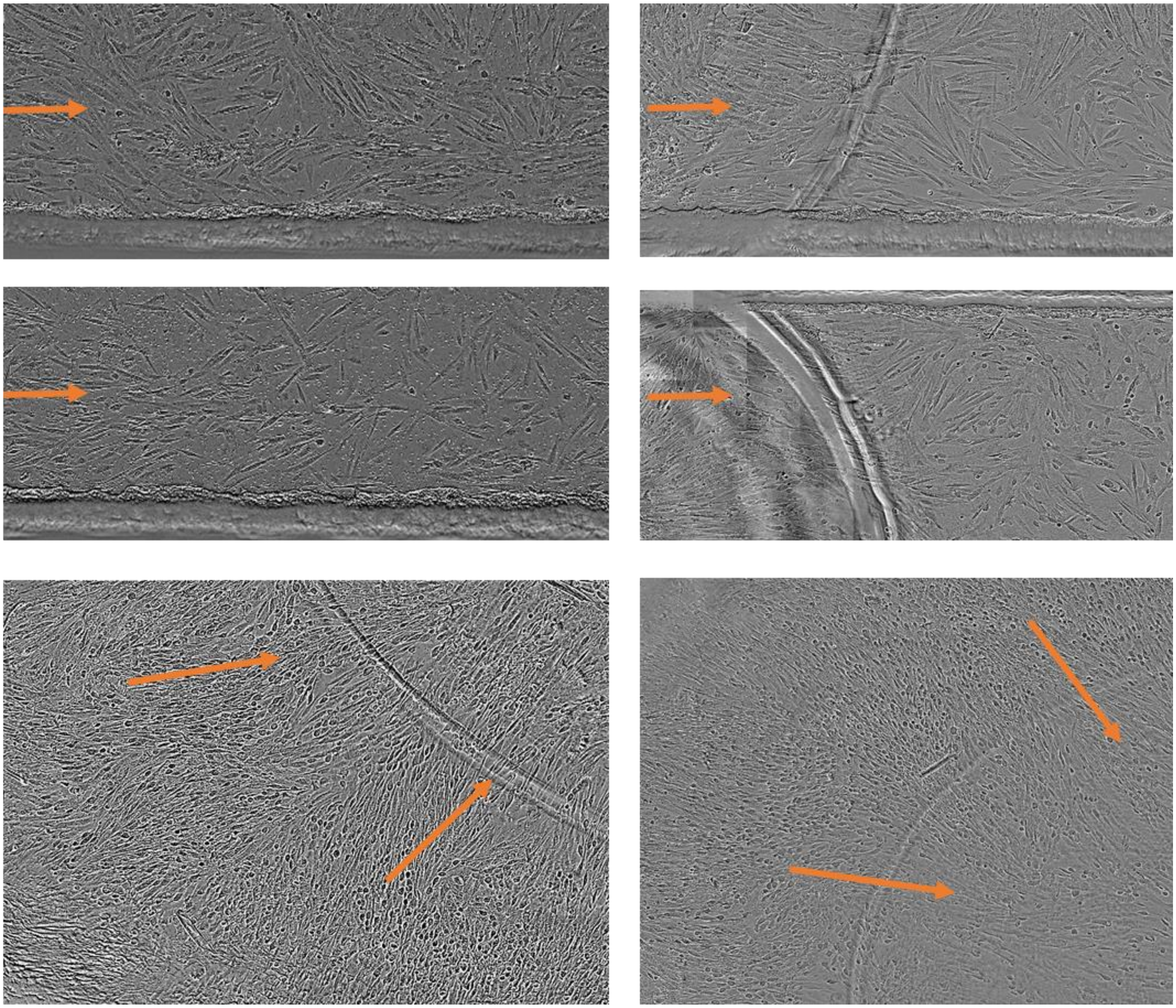

Fig. S 5: Phase contrast images of HLECp2 cells cultivated under flow (orange arrow) and used for analysis of the orientation.

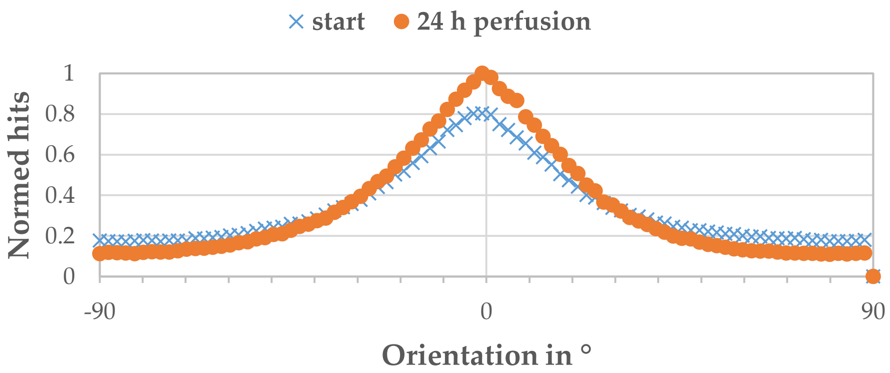

Fig. S 6: Normalized histogram of HLEC cells cultivated under flow at the beginning and end of the experiment
